# Patient Satisfaction with Clinical Pharmacist Communication and Its Association with Treatment-Related Problems: A Cross-Sectional Study in Jordanian Public Hospitals

**DOI:** 10.3390/healthcare14091176

**Published:** 2026-04-28

**Authors:** Mohammad Ali AL-Qarni, Ahmet Sami Bosnak, Esra’ O. Taybeh

**Affiliations:** 1 Department of Pharmaceutical Sciences, Faculty of Pharmacy, Cyprus International University, North Cyprus, Mersin 10, 99258 Nicosia, Türkiye; 2 Department of Clinical Pharmacy, Faculty of Pharmacy, Cyprus International University, North Cyprus, Mersin 10, 99258 Nicosia, Türkiye; abosnak@ciu.edu.tr; 3 Department of Applied Pharmaceutical Sciences and Clinical Pharmacy, Faculty of Pharmacy, Isra University, Amman 11622, Jordan; esra.taybeh@iu.edu.jo

**Keywords:** clinical pharmacist communication, patient satisfaction, treatment-related problem, patient knowledge, patient adherence, medication errors

## Abstract

**Background:** Treatment-related problems (TRPs), including patient adherence, knowledge, and medication errors, affect hospitalized patients’ outcomes worldwide. Effective clinical pharmacist communication is essential for proper patient counselling and medication safety. However, the effect of clinical pharmacist communication skills on TRPs has not been adequately studied. **Objective:** This study aimed to assess patients’ satisfaction with clinical pharmacists’ communication components (appropriate timing, language, and empathy) and examine their association with TRPs, including patient adherence, knowledge, and medication errors. **Method:** A cross-sectional study was conducted in two Jordanian public hospitals between October 2023 and April 2024 through a structured questionnaire to assess patients’ satisfaction, adherence, and knowledge. Medication errors were detected in collaboration with other healthcare providers through reviewing medical records and direct patient assessment based on the American Society of Health-System Pharmacists classification. Data analysis was conducted using SPSS version 27. **Results:** A total of 613 adult inpatients were included. Overall, 82.1% (n = 503) of patients were satisfied with the clinical pharmacist communication, 75.5% (n = 463) showed good adherence, and 76% (n = 466) showed good knowledge. A total of 42.4% of medication errors (n = 260) were identified. Errors with harm or fatalities were not observed. The regression results showed that the overall satisfaction level was positively associated with patients’ adherence rate (B = 0.59; *p* < 0.001) and patients’ knowledge (B = 0.978; *p* < 0.001) and negatively associated with the number of medication errors (B = −0.024; *p* < 0.001). **Conclusions:** Clear, timely, and empathetic communication by clinical pharmacists was associated with patient satisfaction and was linked to better patient adherence and knowledge and fewer medication errors. Improving communication skills among clinical pharmacists could be a practical way to reduce TRPs.

## 1. Introduction

Treatment-related problems (TRPs) are defined as events that occur during healthcare delivery and interfere with the achievement of treatment objectives [1]. The most frequent TRP domains are patient knowledge, adherence, and medication errors [1,2]. Medication errors may be associated with factors related to adverse hospital environments, poor communication, and personal factors [3]. Proper communication helps clinical pharmacists in building trust with patients and enhances medication safety [4,5]. The World Health Organization has emphasized the importance of medication safety and highlighted errors related to healthcare providers and their practices, which can affect the treatment strategy and jeopardize patient safety [6]. Proper communication is considered an integral part of an efficient healthcare plan, which is the factor that helps to achieve medication safety [7,8]. Due to the fact that efficient communication is significant, many healthcare institutions have been using patient satisfaction to evaluate communication skills and pharmaceutical care services [9,10].

Many studies have indicated the relationship between communication failure and patient safety [11]. A case study performed in Patan Hospital identified that insufficient communication led to medication errors [12]. Similarly, an Australian retrospective study found that 94.8% of medication errors were linked to insufficient communication practices [13]. Additionally, a study on 250 hypertensive patients indicated that satisfaction with communication was associated with both self-care and treatment adherence [14]. However, it is necessary to consider patients’ educational and cultural backgrounds during communication, which enhances effective conversation, patient confidence, and understanding of the treatment plan [15].

Furthermore, effective communication may contribute to medication safety by providing patients with sufficient information and adequate time for consultation [16]. A study in Taiwan of admitted patients found that those who received more medication education reported higher satisfaction [17]. Patient adherence has also been linked to patient satisfaction. For example, a longitudinal study among patients using antidepressants showed that satisfaction with communication-related factors, including adequate time, empathy, and clear language, provided by healthcare professionals to explain treatment, was linked to better patient adherence [18]. In Jordan, a previous study in the community pharmacy setting also demonstrated relatively positive rates of patient satisfaction with pharmaceutical services, which confirms the importance of patient satisfaction as an indicator of care quality [19].

The major concern of the current healthcare literature has focused on the types, the severity, and the frequency of medication errors globally and particularly in Jordan [20,21]. However, less attention has been paid to patients’ satisfaction with the key elements of clinical pharmacist communication, particularly timeliness, clarity of language, and empathy, and the relationship between these elements and TRPs, including patient adherence, knowledge, and medication errors. Therefore, this study aimed to assess patients’ satisfaction with clinical pharmacists’ communication components (appropriate timing, language, and empathy) and examine their association with TRPs, including patient adherence, knowledge, and medication errors in two public hospitals in Jordan.

## 2. Materials and Methods

### 2.1. Study Design and Setting

A cross-sectional study was conducted on adult inpatients at two public hospitals in Jordan between October 2023 and April 2024 to assess patient satisfaction with clinical pharmacist communication skills, patient adherence, knowledge, and medication errors.

### 2.2. Study Population, Eligibility, and Sampling Technique

#### 2.2.1. Study Population

The study sample included all adult inpatients admitted during the study period into the two selected public hospitals.

We clarified that participants were recruited from multiple inpatient departments (e.g., internal medicine, surgery, and other general wards), reflecting routine hospital admissions rather than a single specialty unit.

#### 2.2.2. Eligibility (Inclusion and Exclusion Criteria)

Eligible participants in this study were adult inpatients aged 18 years and older. They were able to understand the objectives of this study, provide informed consent, and be hospitalized for at least 3 days. We excluded patients admitted to the Intensive Care Unit (ICU), Coronary Care Unit (CCU), Post-Anesthesia Care Unit (PACU), operating rooms, delivery rooms, and emergency units, patients with cognitive impairments and psychiatric disorders, outpatient clinic visitors, and patients staying in the hospital for two days or less.

#### 2.2.3. Sampling Technique

The sample size of this study was estimated using the RAOSOFT sample size calculator (Raosoft, Inc., Seattle, WA, USA), assuming a 95% confidence level, a 5% margin of error, and a conservative estimate of *p* = 0.5 (50% response distribution). So, 384 was the minimum sample size. Convenience sampling was used in the present study for practical reasons, as patient availability depends on ward admission, status, length of stay, and clinical stability. Although a convenience sampling method was used, participants were selected according to predefined inclusion and exclusion criteria, ensuring sample eligibility and suitability for this study’s objectives. Furthermore, the sample size was determined using a standardized statistical calculator, and a large number of patients were included to minimize potential bias.

### 2.3. Data Collection Instruments

#### 2.3.1. Patients’ Satisfaction, Adherence, and Knowledge Instrument

Data were collected using a structured questionnaire that comprised three sections. The first section contained the basic socio-demographic data of the participants (gender, educational level, age, nationality, and marital status) and medical information (number of medications per day and patient hospitalization rate). The second section assessed “Patients’ Satisfaction with Clinical Pharmacist Communication Skills.” This section was adapted from previously published studies evaluating patient satisfaction with pharmacist communication [22,23,24,25]. It included 14 items across three domains: (a) patient satisfaction with clinical pharmacists’ contact time (4 questions), (b) patient satisfaction with the language of communication employed by the clinical pharmacist (4 questions), and (c) satisfaction of patients with the empathetic approach of clinical pharmacists (6 questions). The responses were rated using a 5-point Likert scale (1 = strongly disagree to 5 = strongly agree). Overall satisfaction was defined as ≥60% of the maximum score, corresponding to a mean of ≥3/5 [26].

The third section assessed patient adherence (4 items) and patient knowledge (5 items), adapted from a previously established TRPs framework developed by AbuRuz et al. [1]. Among the many diverse assessment frameworks that focus primarily on the pharmacological aspect, the TRPs framework was chosen because it can measure several patient-centered medication-related outcomes at the same time, such as adherence and knowledge, which align with the purpose of the current study. The responses were measured using the 5-point Likert scale (1 = never to 5 = always). According to the literature [27,28], scores were dichotomized and analyzed. Adherence was defined as ≥80% of the maximum, equivalent to a mean of ≥4/5 [27], and sufficient knowledge as ≥60% of the maximum, equivalent to a mean of ≥3/5 [28].

#### 2.3.2. Medication Errors Assessment Sheet

All medication errors were detected in collaborative pharmaceutical practice with other healthcare providers through direct patient observation. The differentiation among medication error types was performed by referring to the explanatory definitions of medication error types in the American Society of Health-System Pharmacists (ASHP) classification [29]. The error severity was categorized according to the National Coordinating Council for Reporting and Prevention (NCC-MERP), from A to I. The categories were as follows: (A) indicated conditions with potential occurrence of error ability; (B–D) errors that reached patients with no harm; (E–H) showed errors with temporary or permanent harm; and category (I) represented fatal errors [30].

The patients filled out the questionnaires themselves; those who were unable to participate were excluded, and no other parties besides the patients were relied upon to answer in order to reflect the patients’ true experiences and ensure the accuracy of the assessment.

#### 2.3.3. Medical Record

The electronic medical record of each participating patient was reviewed to verify the accuracy of the patient’s demographic data, collect additional data from medication orders, and extract medication administration records, medical history, medical notes, laboratory results, and other related medical data.

## 3. Validation and Reliability

The face and content validity of the structured questionnaire were assessed by a panel of five clinical pharmacy experts for the relevance, clarity, and alignment of the questions with this study’s objectives. The questionnaire was translated from English into Arabic and then back-translated into English by bilingual experts to ensure accuracy. A pilot study was conducted with 25 patients to evaluate the clarity, feasibility, and timing of the questionnaire and the data collection process before the commencement of the main study. Feedback led to minor wording changes. Pilot data were not included in the final analysis. Cronbach’s alpha (α) was used to assess the internal consistency of the questionnaire. The Cronbach’s alpha (α) scale for patients’ satisfaction was 0.895, for patient adherence, it was 0.815, and for patient knowledge, it was 0.827.

## 4. Data Collection Procedure

Data collection was conducted using consistent, structured procedures, in which inpatients were selected from both public hospitals during the study period according to pre-defined inclusion and exclusion criteria. All their information, such as age, willingness to participate, and medical history, was verified through direct review of medical records.

Before distributing the questionnaires for patients to complete, we explained the research aims and objectives. After obtaining the patient’s written consent, the participants voluntarily initiated the process, having full assurance of secrecy and the right to withdraw at any time without affecting their therapeutic plans. The patients filled out the questionnaires themselves. The medical records were reviewed with prior ethical approval from both the hospitals and the patients, with a strong emphasis on maintaining confidentiality and privacy.

The type of medication errors was evaluated based on the ASHP classification, while the severity of medication errors was assessed using the NCCMERP index from A to I, noting that the assessment was done in collaborative pharmaceutical practice with other healthcare providers. This dual assessment reflects lower bias and ensures the accuracy of study outcomes.

### 4.1. Ethical Consideration

Ethical approval was granted by the Jordanian Ministry of Health and both the hospitals’ Institutional Review Boards (Approval No. 7324 and No. 5285). The original author of the third section, the structured questionnaire (patient adherence and knowledge questions), was contacted via email to obtain formal permission. All participants reviewed the written consent form and approved participation in this study. The consent form contained the study title, the researcher’s name, the research objective, a guarantee to participants that participation is voluntary, no interference with their medical care plan, and confidentiality and anonymity of the information provided, which were all kept on a secure, password-protected computer.

### 4.2. Statistical Analysis

The data were analyzed using IBM SPSS Statistics (version 27.0). Continuous variables were summarized as means ± SD and categorical variables as frequencies (n) and percentages (%). Bivariate relationships between overall patient satisfaction and each outcome were investigated using Pearson correlation. Multiple linear regression models were conducted separately for patient adherence, knowledge, and the number of medication errors per patient. The predictor variables were gender, education level, age, number of medications/days, length of stay, marital status, and overall patient satisfaction. A backward elimination (removal) procedure was used, and only predictors retained in the final models were reported. The statistical significance was set at *p* < 0.05.

## 5. Results

A total of 641 eligible individuals were approached, 613 of whom agreed to participate in this study, representing a response rate of 96%. Among 613 participants, more than half the participants were males (60.5%). Approximately one-third held a bachelor’s degree, and the largest age group was “18–27 years”. The majority of the patients used 3–6 medications, and the maximum length of patients’ hospital stay ranged between 4 and 10 days. Mostly, the participants were Jordanians. The full demographic characteristics are displayed in Table 1.

Overall, 82.1% (n = 503) of patients were satisfied with the clinical pharmacist’s communication. In general, satisfaction was high across the three domains, with the clinical pharmacist’s contact time achieving the highest mean score, followed by language of communication and empathetic approach. Table 2 shows the detailed item-level results.

Approximately three-quarters of patients were identified as adherent (75.5%; n = 463). The item-level adherence was generally high, as shown in Table 3.

Overall, 76% (n = 466) of patients were classified as having good knowledge. The detailed knowledge-related items are displayed in Table 4.

A total of 260 medication problems were identified (42.4%), averaging approximately one medication problem per patient. The most common problems were related to compliance, followed by wrong-time errors. Most (98.9%; n = 257) were categorized between A and C, and 1.1% (n = 3) were categorized as category D, without any harm. Meanwhile, harmful or potentially fatal medication errors were not observed in this study. The full data are presented in Table 5.

Pearson correlation analysis showed that all communication domains (contact time, language, and empathy) were significantly positively correlated with adherence and patient knowledge and significantly negatively correlated with the number of medication errors. The detailed coefficients are shown in Table 6.

Multiple linear regression analysis identified significant predictor factors of the main outcome variables. Higher patient satisfaction was associated with higher patient adherence (B = 0.59; *p* < 0.001) and higher patient knowledge (B = 0.978; *p* < 0.001). For the medication errors model, older age (B = 0.056; *p* < 0.001) and a higher number of daily medications (B = 0.244; *p* < 0.001) were linked with more medication errors. In contrast, higher patient satisfaction was associated with fewer medication errors (B = −0.024; *p* < 0.001). Table 7 shows the full regression results.

## 6. Discussion

This study presents insights from two major public hospitals in Jordan into patients’ satisfaction with clinical pharmacists’ communication during admission and how this satisfaction shapes key treatment outcomes. Across more than 600 inpatients, the findings showed a consistent pattern: patients who demonstrated better communication through regular contact time, clear language, and a more empathetic approach were also more likely to be adherent, knowledgeable, and to have fewer medication problems. This emphasizes that communication is a supportive skill for clinical pharmacists in ensuring patient safety.

The observed associations between communication quality and patient outcomes can be interpreted through several behavioral and cognitive mechanisms. Effective communication—particularly when characterized by clarity, adequate time, and empathy—likely enhances patients’ comprehension of medication instructions, reduces uncertainty, and strengthens trust in healthcare providers. These factors are central to established health behavior models, where improved understanding and perceived support increase adherence and engagement in care. Conversely, suboptimal communication may contribute to misunderstanding, medication misuse, and reduced motivation to follow therapeutic recommendations, thereby increasing the likelihood of treatment-related problems [14,31].

The majority of patients were highly satisfied with clinical pharmacists’ communication, particularly the timing, clarity of language, and empathetic approach. These findings align with the higher satisfaction scores noted in previous studies [32,33,34,35]. During hospitalization, communication enhanced understanding of medication-related information and elevated overall patient satisfaction [36].

For example, in Jordan, Basheti et al. [37] observed that medication review services were highly satisfactory to patients, which aligns with our findings highlighting the importance of proper communication in effective medication review. However, based on our findings, the average patient satisfaction levels were higher than those reported in Naser and Sbeat’s study [19]. Potentially, this nuance was explained by the nature of the measurement domains utilized. Our study primarily focused on communication components, including time, language, and empathy, whereas their study highlighted organizational and process dimensions, such as medication handling, setting, dispensing, and other services [19]. Furthermore, our result is consistent with previous research indicating that interpersonal relationships play a major role in patient satisfaction with pharmaceutical services, underscoring the importance of effective communication in clinical pharmacy practice [38]. This study demonstrates patients’ satisfaction with the clinical pharmacist’s capability who ensures regular daily interaction.

This result was found to be consistent with the outcomes reported in Hasen and Negeso’s study [22], which stated that satisfaction with the time spent with the pharmacist was rated from satisfied to very satisfied. This indicates that adequate contact time enhances communication and patient engagement. In comparison with Ali et al. [39], the study reported that 53.8% of patients were displeased with pharmacists’ patient contact time, which may be explained by differences in data collection instruments and settings. The communication language was also significant for patient satisfaction, as higher satisfaction was observed when clinical pharmacists applied clear, simple, and familiar language. In line with previous findings, most patients were satisfied with the language of communication due to its simplicity and clarity [39,40].

Furthermore, patients expressed satisfaction with the empathetic attitude of clinical pharmacists, which aligns with Chevalier et al.’s study, in which most patients were highly satisfied with pharmacists’ communication behaviors [41].

About three-quarters of patients had good knowledge of their disease or medications, suggesting that communication with clinical pharmacists and other healthcare providers provides better access to relevant information. Health awareness is growing among patients due to the extensive use of the internet [42], improved communication between clinicians and patients [43], and stronger social support [44]. Such a pattern was observed in Jordanian research evaluating health knowledge and awareness of proper antibiotic use and antibiotic resistance among Jordanian patients, in which patient awareness and knowledge noticeably increased over time [45].

The number of medication errors in this study was low. With one problem reported per patient, which is noticeably lower than reports from Jordanian hemodialysis cohorts (~7 TRPs/patient) [46] and chronic-disease outpatients (with means of 7.4 and 11.2 TRPs/patient) [47,48]. This finding should be interpreted cautiously. This finding may not necessarily indicate a lower true incidence but could reflect methodological differences, including reliance on observational and record-based detection methods and the exclusion of high-risk units, such as intensive care settings. Additionally, the integration of clinical pharmacists into patient care processes may have contributed to early identification and mitigation of potential errors, thereby reducing their recorded occurrence.

The regression results showed that older age and a greater number of daily medications predicted a higher number of medication errors, consistent with findings in Jordanian dialysis populations, indicating an association between problem burden and age and number of medications [46]. While aging brings multi-morbidity and renal pharmacodynamic–pharmacokinetic alterations, high pill burden is linked to the chance of medication interactions and cognitive load. These challenges can also impair the use of routine medications and self-management [49,50].

Consistent with our findings, overall patient satisfaction with pharmacist communication was associated with better adherence [51,52]. Trust, specifically, decreases resistance to treatment recommendations, while clarity of instructions eradicates practical barriers to adherence, such as misunderstandings about dosing, timing, and possible side effects [53]. Evidence from the global literature reinforces this relationship, emphasizing that effective communication is the cornerstone of treatment adherence and medication safety. For example, a Vietnamese study observed that the direction of the organized pharmacist intervention enhanced medication adherence and glycemic control in diabetic patients [54]. A cohort study in Pakistan observed that poor pharmaceutical consultation was linked to low patient satisfaction and restricted access to patient education and counseling, eventually affecting medication adherence. This aligns with our study’s finding that overall patient satisfaction is significantly associated with higher patient knowledge [55].

The relatively elevated patient satisfaction levels identified in this study may be affected by contextual factors unique to the study environment. In public hospitals, where healthcare systems often lack sufficient resources, patients may care more about how they are treated by staff, such as how well they communicate and how much they care. This could make pharmacist–patient interactions seem more important than they are in healthcare systems that rely more on technology, thereby strengthening those connections.

This study has some limitations that should be considered. First, the cross-sectional design precludes causal inference; therefore, the observed relationships between clinical pharmacist communication and TRPs should be interpreted only as associations. Second, the use of convenience sampling may introduce selection bias and limit the representativeness of the study population. In addition, participants were recruited from general inpatient settings without stratification by hospital department, and prior exposure to patient education programs was not assessed, which may act as unmeasured confounders. Thirdly, adherence and knowledge were assessed through self-report instruments, which are susceptible to recall bias and social desirability bias. Furthermore, the employment of analogous Likert-scale measures across multiple constructs introduces the potential for shared method variance. Fourth, although medication errors were classified using established frameworks, inter-rater reliability was not formally evaluated, and variability in clinical pharmacists’ communication training was not assessed, potentially affecting the consistency of communication practices. Finally, as this study was conducted in two public hospitals in Jordan, the generalizability of the findings to other healthcare settings may be limited.

Multicenter, longitudinal, and interventional research should be considered in future studies to determine whether enhancing the pharmacist’s communication skills results in a quantifiable change in treatment-related outcomes.

## 7. Conclusions

Patient satisfaction with clinical pharmacists’ communication was high and positively associated with patients’ knowledge and adherence, whereas satisfaction was inversely associated with the number of medication errors. Clear, consistent, and empathetic communication is critically important for safe and optimal care. Practicing pharmacists should be encouraged to develop their professional communication skills in healthcare settings.

## Figures and Tables

**Table 1:** Demographic and medical data of participants (N = 613).

Variable	Freq.	Percentage%
**Gender**		
**Male**	371	60.5%
**Female**	242	39.5%
**Educational level**		
**Primary education**	55	9%
**Secondary education**	80	13.1%
**Community college (Diploma)**	171	27.9%
**Bachelor’s**	224	36.5%
**Master’s and Ph.D.**	83	13.5%
**Age (years)**		
**18–27**	141	23%
**28–37**	120	19.6%
**38–47**	89	14.5%
**48–57**	110	17.9%
**58–67**	106	17.3%
**≥68**	47	7.7%
**Study facility**		
**Hospital 1**	291	47.5%
**Hospital 2**	322	52.5%
**Number of medications/days**		
**Less than 3**	119	19.4%
**3–6**	482	78.6%
**More than 6**	12	2%
**Length of stay**		
**3 days**	192	31.3%
**4 days-10 days**	290	47.3%
**More than 10 days**	131	21.4%
**Nationality**		
**Jordanian**	607	99.0%
**Others**	6	1.0%
**Marital status**		
**Single**	95	15.5%
**Married**	496	80.9%
**Widow**	12	2%
**Divorced**	10	1.6%

**Table 2:** Patient satisfaction with clinical pharmacists’ communication skills (N = 613).

Items	Mean ± SD Patient Satisfied n (%)
Contact time	
1. The clinical pharmacist gives you sufficient time during the communication.	3.80 ± 1.38
2. The clinical pharmacist is accessible and keeps in touch with you every day.	3.72 ± 1.26
3. The clinical pharmacist communicates with you at different times, even in a crowded environment.	3.84 ± 1.34
4. The clinical pharmacist sets a specific time for you and does not exceed it, even if you need more time to talk *	3.74 ± 1.30
**Overall Patient Satisfaction with Contact Time**	**3.77 ± 0.78**
Language of communication	
1. When communicating with you, the clinical pharmacist uses medical terms that are difficult to understand *	3.72 ± 1.26
2. When speaking with you, the clinical pharmacist uses a language or dialect that is different from your dialect or language. *	3.81 ± 1.25
3. The clinical pharmacist uses language that raises anxiety and instills fear in you. *	3.77 ± 1.29
4. The clinical pharmacist uses the language of mutual dialogue with you.	3.76 ± 1.31
**Overall Patient Satisfaction with Language of Communication**	**3.76 ± 0.68**
The empathic approach	
1. The clinical pharmacist cares, listens, and shows attention when you speak.	3.55 ± 1.42
2. The clinical pharmacist reassures, supports, and relieves your psychological pressures.	3.60 ± 1.42
3. The clinical pharmacist deals with you in a friendly way, such as smiling and looking towards you.	3.71 ± 1.45
4. The clinical pharmacist handles your condition in an emotional way and makes you feel close to him/her.	3.58 ± 1.49
5. The clinical pharmacist tries to know all your concerns and problems to deal with them.	3.60 ± 1.45
6. The clinical pharmacist encourages you to provide all the information related to your health condition.	3.53 ± 1.36
**Overall Patient Satisfaction with Empathic Approach**	**3.60 ± 0.94**
**Overall Satisfaction across All Domains**	**3.70 ± 0.77** **503 (82.1%)**

* Reverse coding; ≥60% satisfaction.

**Table 3:** Patient adherence assessment items scores (N = 613).

Items	Mean ± SD	Adherent Patients *
Do you forget taking your medication? *	4.07 ± 1.38	
Do you run out of your medications? *	4.23 ± 1.25	
Do you skip taking your medication if you feel better or worse? *	4.41 ± 1.13	
Do you follow your clinical pharmacist’s advice about your diet, exercise, smoking, etc.?	4.33 ± 1.24	
Overall Patient Adherence Score	4.26 ± 0.71	463 (75.5%)

* Reverse coding; patients with ≥80% adherence score were considered adherent.

**Table 4:** Patient knowledge assessment items scores (N = 613).

Items	Mean ± SD	Patients with Good Knowledge
Do you have information about your illness and medical condition?	3.53 ± 1.3	
Do you Know when and how to take your medication?	3.57 ± 1.2	
Do you Know the instructions for your medication?	3.55 ± 1.34	
Do you Know what foods to avoid?	3.59 ± 1.33	
Do you know why you are taking your medication?	3.52 ± 1.41	
Overall Patient’s Knowledge Score	3.55 ± 0.84	466 (76.0%)

Patients who gained ≥ 60% were considered to have good knowledge.

**Table 5:** Types and number of medication problems (N = 260).

Type of Problem	No# of Problems	Percentage%
**Prescribing**	13	2.1%
**Omission**	0	0.0%
**Wrong time**	49	8.0%
**Unauthorized drug**	0	0.0%
**Improper dose**	0	0.0%
**Wrong dosage form**	0	0.0%
**Wrong drug preparation**	3	0.5%
**Wrong administration technique**	6	1.0%
**Deteriorated drug**	0	0.0%
**Monitoring** **Compliance** **Other**	0 189 0	0% 30.8% 0%
**Total**	260	42.4%

**Table 6:** Pearson correlation between study variables (N = 613).

Variables	1	2	3	4	5
1. Contact time	1				
2. Language	0.854 **	1			
3. Empathic approach	0.884 **	0.741 **	1		
4. Adherence	0.631 **	0.614 **	0.608 **	1	
5. Knowledge	0.899 **	0.849 **	0.842 **	0.632 **	1
6. Medication error	−0.533 **	−0.427 **	−0.525 **	−0.678 **	−0.531 **

** *p*-value < 0.001.

**Table 7:** Results of linear regression analysis of predictive factors affecting treatment-related problems (TRPs).

Factor	B Coefficient	*p*-Value	95.0% CI for B	
			LL	UL
A. Patient adherence				
Overall patient satisfaction	0.590	≤0.001	0.532	0.648
B. Patient knowledge				
Overall patient satisfaction	0.978	≤0.001	0.940	1.015
C. Number of medication errors				
Age (years)	0.056	≤0.001	0.027	0.084
Number of medications/days	0.244	≤0.001	0.134	0.354
Overall patient satisfaction	−0.024	≤0.001	−0.027	−0.020

Separated models were fitted for (A) patient adherence score, (B) patient knowledge score, and (C) number of medication errors per patient using backward elimination (removal method). B = unstandardized regression coefficient; CI = confidence interval; LL = lower limit; UL = upper limit; *p*-values < 0.05.

## Data Availability

The data presented in this study are available upon request from the corresponding author. The data are not publicly available due to privacy and ethical restrictions.
